# Effect of Post-Mixing pH Regulation of a Gelatin–κ-Carrageenan System on the Structure and 3D Printing Performance of Yellow Peach Pulp Gels

**DOI:** 10.3390/gels12060472

**Published:** 2026-05-29

**Authors:** Yidian Li, Yunyi Gong, Xuejiao Wang, Yongshuai Ma, Rui Chai, Zhenna Zhang, Chaofan Guo, Junjie Yi

**Affiliations:** 1 Faculty of Food Science and Engineering, Kunming University of Science and Technology, Kunming 650500, China; liyidian0109@163.com (Y.L.); jocelyn04-02@outlook.com (Y.G.); wangxuejiao173@hotmail.com (X.W.); mays@kust.edu.cn (Y.M.); 20242225034@stu.kust.edu.cn (R.C.); junjieyi@kust.edu.cn (J.Y.); 2 Yunnan Key Laboratory of Plateau Food Advanced Manufacturing, Kunming 650500, China; 3 Yunnan International Joint Laboratory of Green Food Processing, Kunming 650500, China; 4 International Green Food Processing Research and Development Center of Kunming City, Kunming 650500, China; 5 College of Food Science and Engineering, Northwest A&F University, Yangling, Xianyang 712100, China; zn.zhang@nwafu.edu.cn

**Keywords:** gelatin–κ-carrageenan, post-mixing pH regulation, yellow peach pulp gel, 3D food printing, extrusion behavior, printing fidelity

## Abstract

Extrusion-based three-dimensional food printing requires inks that can be smoothly extruded while maintaining sufficient structural stability after deposition. In this study, gelatin and κ-carrageenan were first mixed and then subjected to post-mixing pH regulation before spray drying, producing composite powders with different structural states. These powders were incorporated into yellow peach pulp gels to prepare fruit-based printing inks, and their printing performance, extrusion behavior, mechanical properties, particle-size distribution, and microstructure were systematically evaluated. The results showed that the structural state formed during gelatin–κ-carrageenan powder preparation was closely associated with the extrusion stability and shape retention of the final inks. Among the tested formulations, the ink prepared with gelatin–κ-carrageenan powder pre-regulated to pH 4.0 exhibited the best overall printability. Although its pore-area fidelity was slightly lower than that of the sample pre-regulated to pH 3.5, it produced more stable multilayer cylinders and better-defined lattice structures. In addition, the pH 4.0 sample showed the lowest and most stable extrusion force and the highest Young’s modulus, indicating a favorable balance between extrusion flowability and post-deposition support. Microstructural observations and particle-size analysis suggested that pH regulation altered the aggregation state and local morphology of the gelatin–κ-carrageenan system. Samples prepared at higher pH values tended to form larger and less uniform aggregates, which was unfavorable for stable extrusion and shape retention. Overall, post-mixing pH regulation of gelatin–κ-carrageenan provides a practical strategy for improving the printing-related properties of fruit-based gel inks.

## 1. Introduction

Three-dimensional (3D) food printing, especially extrusion-based printing, has attracted increasing attention as a way to produce personalized foods with complex shapes, tailored nutritional composition, and novel textural properties through layer-by-layer deposition [1,2]. For gel-based inks, successful extrusion printing depends on a suitable balance of rheological properties. The material should show relatively low viscosity under high shear so that it can pass smoothly through the nozzle, while also having sufficient mechanical strength and yield stress to recover after extrusion and maintain the printed shape [3,4]. Among these properties, viscosity is particularly important because it strongly affects flowability, extrusion resistance, filament continuity, and shape retention after deposition. Therefore, both excessively low and excessively high viscosity may reduce print quality: the former can lead to collapse after deposition, whereas the latter may cause unstable flow and discontinuous extrusion. For a given printer and nozzle setup, an appropriate rheological range is therefore required. In line with this view, many food materials have been investigated as printable inks, especially gel-like pastes whose viscoelastic properties can be adjusted over a relatively wide range. Typical examples include fruit- and vegetable-based gels as well as surimi gels, which have often been used as model systems to examine how formulation-dependent changes in viscosity influence printability and structural stability. When an ink combines relatively low viscosity during extrusion with sufficient strength after deposition, extrusion resistance can be reduced while interlayer adhesion and shape retention are maintained, thereby improving printing accuracy and structural stability in gel-like food systems [5].

Proteins such as gelatin and polysaccharides such as κ-carrageenan are widely used in 3D food printing because they are naturally derived, biocompatible, and able to provide adjustable gelling and thickening functions. Gelatin contributes thermoreversible elasticity and hydrogen-bond-mediated networks, whereas κ-carrageenan promotes ionic gelation, shear-thinning behavior, and water retention due to its sulfate groups [6]. However, direct blending of these biopolymers into food matrices such as fruit pulps may increase local flow resistance and structural heterogeneity through physical entanglement, hydrogen bonding, and other weak intermolecular associations, which can reduce extrusion stability and printing fidelity in more complex structures [7]. Complex coacervation provides a useful basis for regulating protein–polysaccharide interactions. It is mainly driven by electrostatic attraction between oppositely charged biopolymers, such as positively charged proteins below their isoelectric point and anionic polysaccharides such as κ-carrageenan, and may lead to phase separation or the formation of associated microaggregates [8,9]. Hydrogen bonding and hydrophobic interactions may also contribute to the stabilization of these structures. Therefore, post-mixing pH regulation of a gelatin–κ-carrageenan system may alter its association state, particle distribution, and local structure, which could further affect extrusion flow and post-deposition support during 3D printing. Previous studies have shown that protein–polysaccharide complexes or coacervate-based microcapsules can improve the rheological behavior and printability of food materials [7,10]. For example, Wang et al. reported that a β-carotene encapsulation system based on complex coacervation improved surimi gel properties and showed potential for 3D printing [11]. However, less attention has been paid to using post-mixing pH regulation of gelatin–κ-carrageenan as a practical strategy to improve the printability of acidic fruit-based gels.

In this study, gelatin and κ-carrageenan were first mixed and then regulated to different pH values before spray drying to obtain composite powders with different structural states. To prepare PKGG inks, freeze-dried yellow-flesh peach powder, water, and either KG or PM were blended at a weight ratio of 8:15:1 (*w*/*w*/*w*), stirred to form a uniform dispersion, and hydrated overnight at 25 °C. This study aimed to determine whether KG pre-regulation could influence the extrusion behavior and 3D printing performance of fruit-based inks. Printing performance was evaluated using lattice and hollow-cylinder models, and the underlying differences were analyzed by rheology, simulated extrusion, Young’s modulus, particle-size distribution, and SEM. Unlike previous studies focusing on gelatin–κ-carrageenan interactions, gelatin–gum Arabic coacervates, or peach-based 3D printing gels, this work links post-mixing pH regulation of gelatin–κ-carrageenan with extrusion stability, mechanical support, and printing fidelity in an acidic fruit pulp system. From an application perspective, the spray-dried KG powder could also be used as a pre-regulated additive-like premix, which may facilitate storage, transportation, and incorporation into fruit-based matrices for larger-scale food processing. This strategy may help develop customized 3D-printed fruit products with controlled shapes, textures, and personalized formulations.

## 2. Results and Discussion

### 2.1. Analysis of 3D-Printed Structures

**Figure 1:**
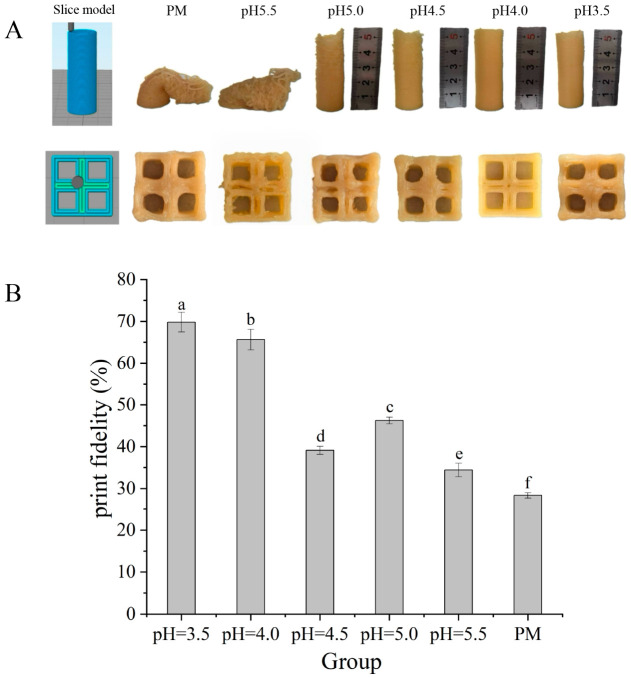
3D printing performance and print fidelity of PKGG under different pH conditions. (**A**) Representative photographs of printed cylindrical (layer-stacking/self-support) and lattice (path fidelity) constructs prepared at various pH values. (**B**) Quantitative comparison of print fidelity, where higher values indicate closer agreement with the designed geometry. Data are presented as means ± standard deviation from three independent trials (*n* = 3). Different lowercase letters indicate statistically significant differences between groups (*p* < 0.05). Under the tested conditions, the pH 4.0 sample showed relatively stable stacking performance and shape retention.

In this study, two representative geometries, a hollow cylinder and a lattice, were used to evaluate printing performance from complementary perspectives. The hollow-cylinder model mainly reflects multilayer stacking and self-supporting ability, as it is sensitive to retained height, sidewall straightness, and resistance to slumping or buckling during deposition [12]. In contrast, the lattice structure contains intersecting filaments and open pores, and is therefore useful for assessing filament continuity, edge definition, junction stability, and path accuracy [13]. As shown in Figure 1A, clear differences were observed among the printed samples. The pH 4.0 sample showed the most stable hollow-cylinder structure, with the greatest retained height, relatively straight sidewalls, and limited visible deformation. Its lattice structure also showed continuous filaments, clearer intersections, and more regular pores, suggesting stable extrusion and deposition under the selected conditions. The pH 3.5 sample could still form a recognizable lattice structure, but its hollow cylinder showed lower retained height and partial deformation, indicating insufficient multilayer support. Samples prepared at pH 4.5 and above showed poorer printing quality, including broadened filaments, merged adjacent lines, incomplete pores, and reduced dimensional stability. The pH 5.5 and PM samples showed particularly poor shape retention, with both cylinder and lattice structures close to collapse. These observations indicate that pH 4.0 provided the best overall printing performance when filament definition, lattice formation, and multilayer self-support were considered together.

The quantitative printing fidelity results in Figure 1B were generally consistent with the visual observations in Figure 1A, but should be interpreted together with the hollow-cylinder printing results. Significant differences were observed among the PKGG samples (*p* < 0.05), and pore-area fidelity decreased with increasing pre-regulation pH. The pH 3.5 and pH 4.0 samples showed the highest fidelity values, approximately 70% and 65%, respectively. Although pH 3.5 showed slightly better pore-area agreement in the lattice model, visual inspection and hollow-cylinder printing indicated that pH 4.0 had better filament definition, deposition stability, and shape retention. Therefore, pH 4.0 was considered to have the best overall printability. At pH 4.5 and pH 5.0, fidelity decreased to approximately 40–45%, while pH 5.5 and PM showed the lowest values, below 30%, consistent with their severe deformation. Although the maximum fidelity was moderate, it was sufficient to maintain basic structural integrity under the present conditions, while further optimization would be needed for more complex 3D food designs.

The pH-dependent printing behavior may be related to differences in the structural state formed during pH regulation of the gelatin–κ-carrageenan system before incorporation into the yellow peach pulp matrix. However, because the final pH values of the formulated PKGG inks were not directly measured, the printing behavior should not be attributed solely to the initial charge state of KG. Instead, it is more reasonable to interpret the results as the combined effect of the pre-regulated KG structure and the final fruit-matrix environment, including possible contributions from fruit-derived organic acids, intrinsic pectin, and interactions among these components. Under the present conditions, pH 4.0 appeared to provide a favorable balance between extrusion flowability and post-deposition support, whereas higher-pH samples showed poorer filament definition and weaker shape retention, possibly due to less favorable structural organization or local heterogeneity. Similar relationships among material structure, rheological behavior, interlayer stability, and printing fidelity have been reported in previous studies [12], and better-organized gel structures have also been associated with improved extrusion fidelity and morphological stability in 3D food printing [14]. Overall, the pH 4.0 treatment produced the most stable and well-defined cylinder and lattice structures among the tested groups, indicating that this condition provided a favorable balance between extrusion flowability and post-deposition support under the present conditions. Despite the maximum printing fidelity being around 65%, which is moderate, this was sufficient under the current conditions to maintain the overall structural integrity of the printed cylinders and lattice patterns. This level of fidelity supports basic 3D food printing applications. For more complex or precision-oriented designs, further optimization of the material formulation and printing parameters could be explored in future studies to enhance fidelity.

### 2.2. Analysis of Rheological Properties

**Figure 2:**
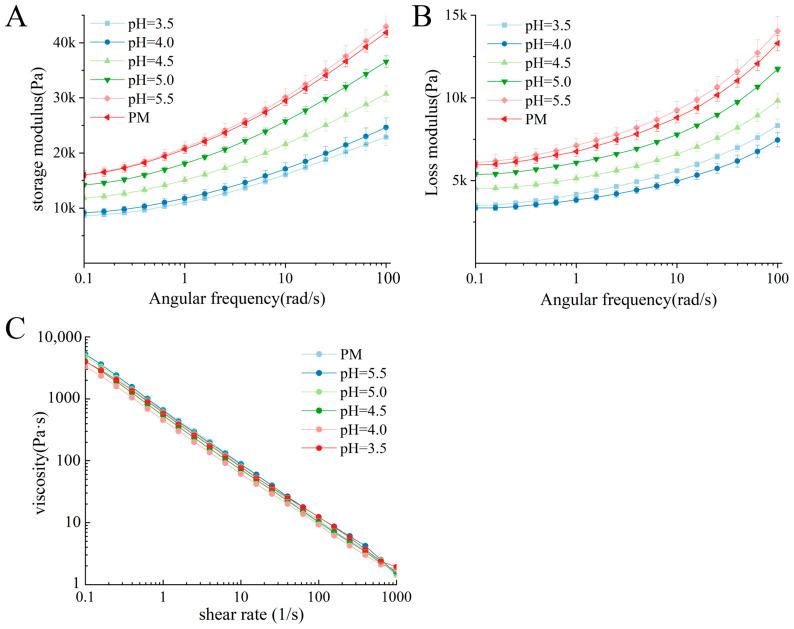
Rheological properties of PKGG under different pH conditions. (**A**) Storage modulus (G′) as a function of angular frequency; (**B**) Loss modulus (G″) as a function of angular frequency; (**C**) Viscosity as a function of shear rate.

Rheological properties, especially storage modulus (G′), loss modulus (G″), and viscosity, are key factors affecting printability, formability, and structural precision in 3D food printing [7,15]. Successful printing requires materials that flow smoothly during extrusion and rapidly recover after deposition to maintain the printed shape [10]. As shown in Figure 2A,B, G′ and G″ of PKGG increased with angular frequency, with G′ remaining higher than G″ throughout the tested range, indicating typical weak-gel behavior dominated by elasticity [16]. The measured moduli were clearly influenced by pH, but their interpretation should also consider the testing conditions. In this study, the rheometer gap was 1.0 mm, while the subsequent particle-size analysis showed that some hydrated KG aggregates could reach the millimeter scale. Although the particle-size analysis was performed on KG rather than the final PKGG printing ink, it suggests that relatively large swollen domains may also exist in the system, especially at higher pH values. When these soft domains approach the scale of the measurement gap, they may partly affect the measured rheological response during oscillatory testing. Therefore, the relatively high moduli of the pH 5.0–5.5 and PM samples should be understood as the combined result of pH-related structural changes and the possible influence of large hydrated domains during measurement.

The G′ values of the pH 3.5 and pH 4.0 samples were relatively close at low and medium frequencies, while the pH 4.0 sample showed slightly higher G′ at higher frequencies. In contrast, the G″ of pH 4.0 was lower than that of pH 3.5 over most of the tested range, indicating lower viscous dissipation while maintaining sufficient elastic response. The pH 5.0–5.5 groups and PM sample generally exhibited higher measured moduli, suggesting stronger resistance to small-amplitude deformation, although this response may also be affected by their more heterogeneous structure. Importantly, higher moduli did not necessarily lead to better printing performance. For the pH 5.5 sample, the more rigid and uneven structure may have increased extrusion resistance and made the deposited filaments less able to relax, fuse, and adapt during layer stacking. In the tall hollow-cylinder model, these effects could amplify small deposition defects and promote tilting or collapse. Therefore, the severe deformation of the pH 5.5 sample in Figure 1A is not inconsistent with its higher measured rheological moduli, but rather suggests that excessive local rigidity and unstable extrusion can compromise overall printing performance [14,17]. This pH-dependent change in viscoelastic behavior was also reflected in the microstructural features discussed later, although the relationship between microstructure and rheological behavior remains inferential. κ-Carrageenan contributes to the gel-like backbone, and when incorporated into yellow peach pulp, it can reinforce the continuous phase, increasing G′ and promoting composite gel formation [18]. The simultaneous increase in G″ indicates enhanced viscous dissipation alongside elastic strengthening, and G′ remained higher than G″ in all groups, showing predominantly solid-like behavior under small-amplitude oscillatory shear.

**Figure 3:**
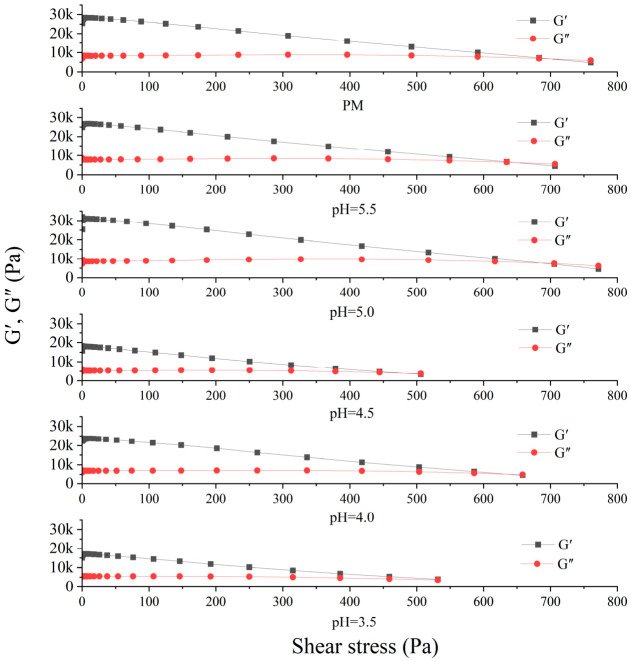
Yield stress analysis of PKGG under different pH conditions. The variation in storage modulus (G′) and loss modulus (G″) with shear stress shows a crossover point where the system transitions from elastic solid-like to viscous liquid-like behavior. The corresponding stress at this intersection is defined as the yield stress (τ_y).

The steady-shear results in Figure 2C showed that all samples exhibited pronounced shear-thinning behavior, with viscosity sharply decreasing as shear rate increased, which is favorable for extrusion printing because it reduces flow resistance during extrusion and allows partial viscosity recovery after deposition. At low shear rates (0.1–1 s^−1^), PM and pH 5.5 showed the highest measured viscosities, while pH 3.5 had the lowest viscosity, indicating that pH affected the internal resistance of the system. However, the relatively high values in the PM and pH 5.5 samples may also be related to their more heterogeneous structure and the presence of large hydrated domains during testing. To further describe the transition from a self-supporting state to a printable flowing state, amplitude sweep results are presented in Figure 3. As shear stress increased, G′ and G″ gradually decreased and eventually crossed over, with the crossover stress (τ_y) representing the measured yield stress. The pH 3.5 sample had the lowest τ_y, while pH 5.0–5.5 and PM showed higher τ_y, suggesting stronger resistance to deformation. Nevertheless, these higher τ_y values may also be partly influenced by the interaction between large swollen domains and the 1.0 mm testing gap. Therefore, the yield stress values should be interpreted together with the particle-size results and the actual printing behavior. The pH 4.0 sample exhibited intermediate τ_y and moderate viscoelasticity, suggesting a better balance between extrusion flow and post-deposition support, consistent with its improved printing stability and shape retention.

### 2.3. Simulated Extrusion Capability of 3D Printing

**Figure 4:**
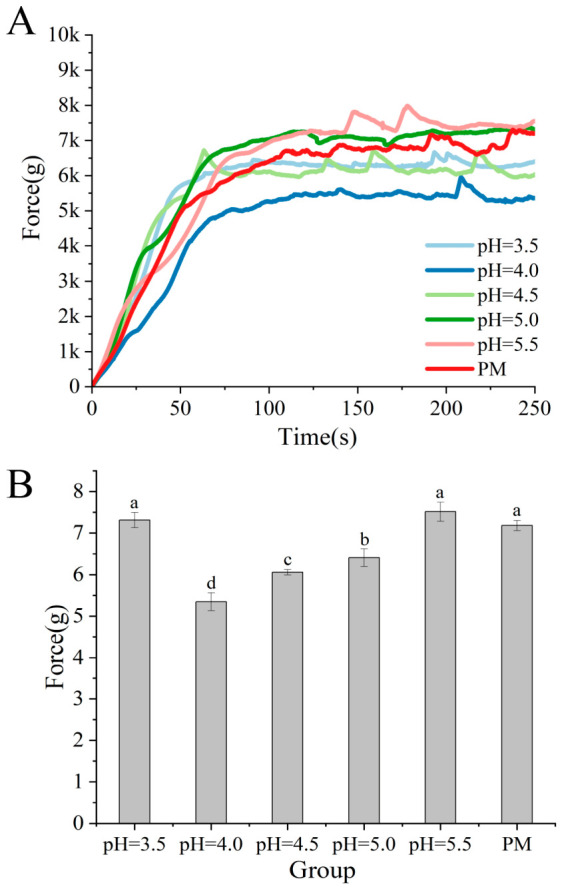
Extrusion performance of PKGG under different pH conditions. (**A**) Force–time curves during the extrusion process. (**B**) Average extrusion force at the steady stage with significance analysis. Data are presented as means ± standard deviation from three independent trials (*n* = 3). Different lowercase letters indicate statistically significant differences between groups (*p* < 0.05). Under the tested conditions, the pH 4.0 sample showed the lowest and most stable extrusion force, suggesting relatively favorable extrusion behavior.

Simulated piston-based extrusion provides a useful indicator of printing performance because it reflects the resistance and stability of material flow through the nozzle under controlled conditions. In extrusion-based 3D food printing, a lower steady-state extrusion force generally indicates lower flow resistance, while a stable force profile is important for maintaining a constant volumetric flow, especially in lattice structures where fine filaments are sensitive to fluctuations. Figure 4 summarizes the extrudability of PKGG at different pH values. The extrusion force increased rapidly during the first 0–50 s, then approached a plateau, reflecting the transition from initial yielding or plug formation to a quasi-steady flow state.

Clear differences among the samples were observed in both the magnitude of the plateau force and the smoothness of the curves. The pH 4.0 formulation showed the lowest plateau force and the smoothest profile, with limited fluctuations, indicating relatively smooth and continuous extrusion and less transient restructuring inside the nozzle. This was consistent with the superior print quality shown in Figure 1A, where the pH 4.0 sample produced the tallest cylinder with straight sidewalls and a lattice structure with continuous filaments and well-defined pores. Lower extrusion resistance and reduced force variability help maintain uninterrupted flow and more consistent filament output, contributing to better stacking stability and higher fidelity of fine structural details. By comparison, the pH 3.5, pH 5.5, and PM samples showed higher steady-state extrusion forces with more obvious oscillations, including periodic spikes. From a processing perspective, these fluctuations indicate less stable extrusion and less consistent volumetric flow, which may lead to local over- or under-deposition along the printing path. In addition to pH-dependent intermolecular interactions, the force fluctuations may also be related to mechanical jamming under confined extrusion. Gelatin and κ-carrageenan are thermoreversible polymers, and after hydration at room temperature, the system may contain swollen soft domains or aggregates rather than a fully homogeneous molecular solution. Although the particle-size data were obtained from KG rather than the final PKGG printing ink, the results discussed in the particle-size analysis section suggest that higher-pH KG systems tended to form larger aggregates. After incorporation into the yellow peach pulp matrix and overnight hydration, such swollen domains may become comparable to the 0.8 mm nozzle opening, especially in the pH 5.0–5.5 and PM groups. A high particle-to-nozzle size ratio could cause squeezing, dragging, temporary obstruction, and sudden release during piston-driven extrusion, thereby contributing to the spikes and fluctuations in the force–time curves. Extrusion force–time curves are highly sensitive to both formulation properties and nozzle constraints, further suggesting that higher extrusion forces and less stable force profiles reduce extrusion robustness under fixed printing conditions [19]. Consistent with this interpretation, the groups with pH ≥ 4.5 showed broadened filaments, merged lines, and incomplete pores in Figure 1A, whereas the pH 5.5 and PM samples were close to collapse, suggesting that deposition control and post-extrusion support were not well balanced. Previous studies have similarly shown that elevated extrusion forces and unstable force profiles increase the risk of intermittent flow and nozzle blockage during extrusion printing [17,20], and over- or under-extrusion are major sources of printing inaccuracy that can be quantified from printed patterns [21].

The quantitative results in Figure 4B further supported the observed trends. The pH 4.0 sample required the lowest steady-state extrusion force, whereas pH 3.5, pH 5.5, and PM showed significantly higher values, with pH 4.5 and 5.0 being intermediate. The difference between pH 4.0 and PM is noteworthy: although both contained the same gelatin and κ-carrageenan concentrations, PM required higher force and showed less stable extrusion, indicating that post-mixing pH regulation, particularly at pH 4.0, produced a formulation more favorable for controlled extrusion and deposition. Because PM remained at its native pH, these differences likely reflect the combined effects of pH-dependent interactions, coacervation-related structural rearrangement, and possible mechanical obstruction during confined extrusion, rather than coacervation alone. These findings also suggest that viscosity alone is insufficient to predict high-quality printing; low and stable extrusion resistance and compatibility between material structure and nozzle geometry are also required. Taken together, the piston-extrusion results indicate that pH 4.0 provided the most favorable extrusion behavior, helping explain its superior printing performance in Figure 1.

### 2.4. Analysis of Young’s Modulus Determination

**Figure 5:**
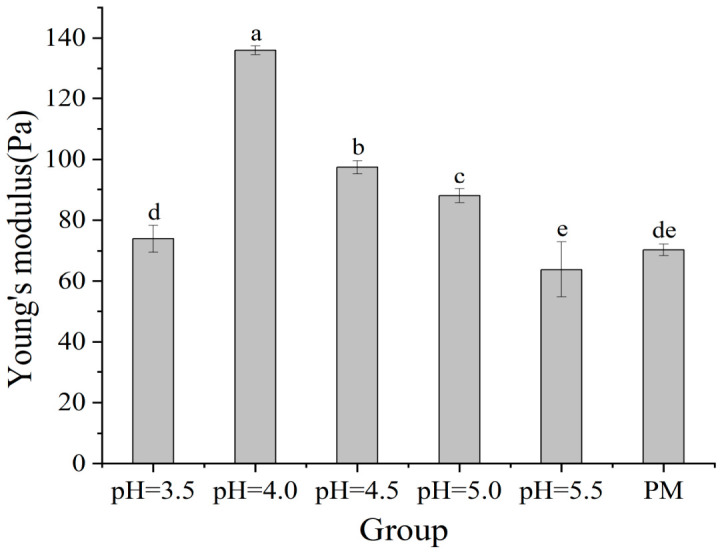
Young’s modulus of PKGG under different pH conditions. Data are presented as means ± standard deviation from three independent trials (*n* = 3). Different lowercase letters indicate statistically significant differences between groups (*p* < 0.05). The pH 4.0 sample showed the highest Young’s modulus, indicating greater resistance to deformation under the tested conditions.

Young’s modulus is an important parameter for evaluating the apparent stiffness and deformation resistance of gel-like materials. In extrusion-based 3D food printing, it is closely related to the ability of deposited filaments to resist deformation under their own weight and under the compression of subsequent layers. Therefore, an appropriate Young’s modulus is beneficial for maintaining layer geometry and reducing slumping or collapse after deposition [22]. Figure 5 shows the pH-dependent changes in the Young’s modulus of PKGG.

The Young’s modulus of PKGG first increased and then decreased with increasing pre-regulation pH, reaching the highest value at pH 4.0, about 140 Pa, which was significantly higher than that of the other groups (*p* < 0.05). In contrast, the pH 5.5 and PM samples showed the lowest values, around 60–70 Pa. This result suggests that the KG system prepared at pH 4.0 contributed more effectively to the mechanical support of the final PKGG ink. From the perspective of printing, the higher modulus of the pH 4.0 sample indicates stronger resistance to small-strain deformation, which is consistent with the better layer stacking, straighter sidewalls, and higher retained height observed in the printed cylinders. By comparison, the lower modulus values of the pH 5.5 and PM samples suggest weaker post-deposition support, which may partly explain their more obvious sagging and collapse during multilayer printing.

The higher Young’s modulus of the pH 4.0 sample may be related to the structural state formed during pH regulation of the gelatin–κ-carrageenan system before incorporation into the yellow peach pulp matrix. During KG preparation, pH 4.0 may favor a suitable degree of association between gelatin and κ-carrageenan, involving electrostatic attraction, hydrogen bonding, and other weak interactions [8]. This interpretation is also supported by the microstructural features discussed later in the microstructural characterization section, although the relationship between microstructure and mechanical behavior remains indirect in the present study. A relatively more continuous and compact microstructure may facilitate load transfer and contribute to higher macroscopic stiffness, which may help explain why the pH 4.0 group showed the highest Young’s modulus.

### 2.5. Microstructural Characterization (SEM) of KG and PKGG

**Figure 6:**
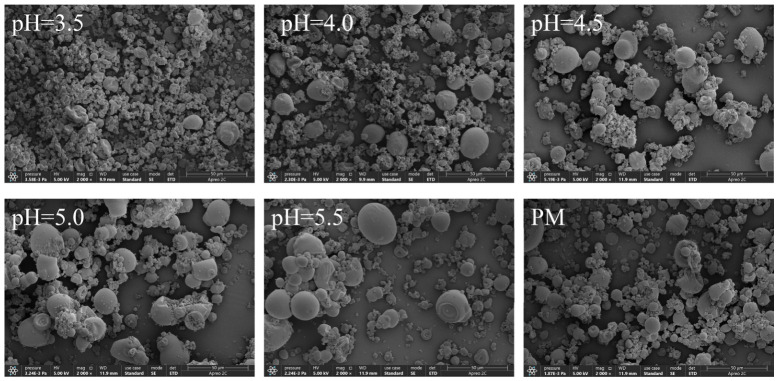
Scanning electron micrographs of KG under different pH conditions at 2000× magnification. The images show visible differences in particle morphology, aggregation state, and surface features among samples prepared at different pH values.

Figure 6 shows the SEM images of KG prepared under different pH conditions, revealing clear pH-dependent changes in particle morphology. At pH 3.5, gelatin is expected to carry a relatively higher positive charge, while κ-carrageenan remains negatively charged, which may favor rapid association between the two biopolymers during pH regulation [6]. SEM images showed many fine particles with some locally compact clusters, suggesting rapid complexation and the formation of non-uniform microaggregates with limited further structural rearrangement.

At pH 4.0–4.5, the microstructure appeared more regular, with more spherical and uniformly distributed particles, smoother surfaces, and lower apparent porosity, suggesting a more moderate balance between complexation and structural rearrangement. As pH increased to 5.0–5.5, approaching the gelatin isoelectric point (≈4.7–5.0), reduced net charge may have weakened electrostatic stabilization and promoted coalescence into larger flocs. SEM images showed enlarged aggregates, looser packing, wrinkled surfaces, and increased porosity, indicating a less uniform structure. Near neutral pH (≈6.0, PM), weaker electrostatic interactions and non-electrostatic forces such as hydrogen bonding and hydrophobic association may have contributed to a more porous architecture. From a printing perspective, a compact microstructure at very low pH does not necessarily correspond to a mechanically favorable printable state. Fine but non-uniform aggregates may reduce deformation tolerance and post-extrusion recovery, impairing layer stacking stability [23]. By contrast, the microstructure at pH 4.0, with relatively regular particles and compact microaggregates, suggests a balanced structural state favorable for continuous extrusion and post-deposition support. This interpretation aligns with previous reports showing that uniform microdomains improve deposition control and structural recovery, whereas excessive flocculation reduces printing resolution and destabilizes fine features [4,24].

**Figure 7:**
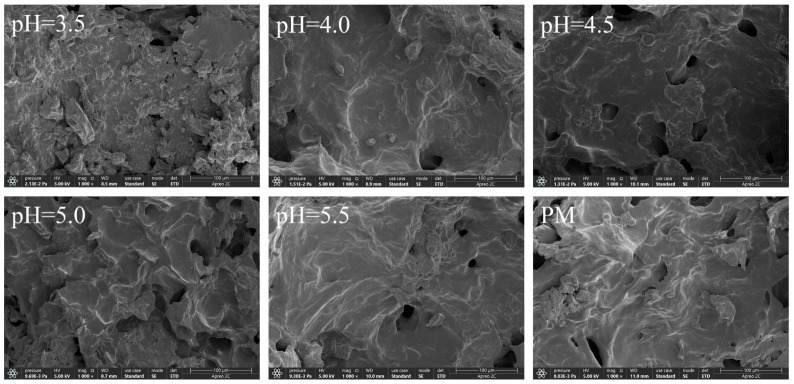
Scanning electron micrographs of PKGG under different pH conditions at 1000× magnification. The images show representative local differences in pore structure and surface morphology among freeze-dried samples prepared at different pH values.

Figure 7 presents representative local SEM images of freeze-dried PKGG samples prepared with KG regulated under different pH conditions, showing differences in pore morphology and surface features. These images should be interpreted as localized observations of the freeze-dried structure rather than direct evidence of the full three-dimensional network in the hydrated printing ink. In samples prepared with KG regulated at pH 3.5, aggregation, irregular fracture surfaces, and disordered porous morphology were observed, suggesting structural heterogeneity that may be unfavorable for stable deposition and mechanical support. When KG was regulated at pH 4.5 and above, larger domains, enlarged pores, thinner or partially collapsed walls, and looser local structures became more apparent, which may be related to reduced charge stabilization and enhanced aggregation during KG formation. The pH 5.5 and PM groups showed more discontinuous pores and wrinkled features, indicating weaker local structural integrity and greater susceptibility to collapse during dehydration. By contrast, the sample prepared with KG regulated at pH 4.0 exhibited a relatively smoother surface and more uniformly distributed local pores, with some fibrous features and interconnected pore-like structures. These observations were broadly consistent with the improved mechanical stability and post-deposition support of this sample during extrusion printing. However, because SEM provides a limited field of view and the samples were freeze-dried before observation, these morphological features should be regarded as indirect evidence rather than definitive proof of macroscopic structural continuity [12,25]. Overall, the local morphology of the sample prepared with KG regulated at pH 4.0 was consistent with its more stable extrusion behavior and better retention of the designed geometry during printing.

### 2.6. Droplet Size Distribution of KG

**Figure 8:**
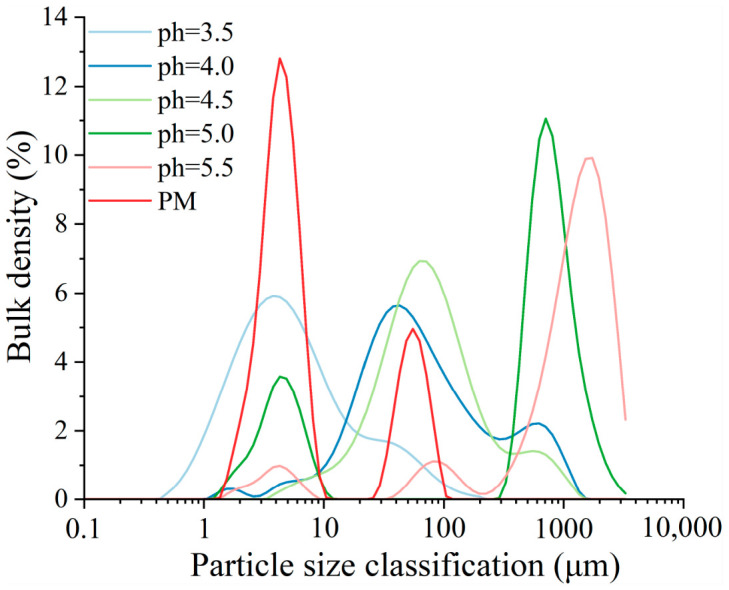
Particle size distribution of KG at different pH levels. The plots show pH-dependent changes in particle aggregation, with intermediate pH (4.0–4.5) producing moderately sized, relatively uniform particles.

As shown in Figure 8, changes in pH markedly altered the particle-size distribution of the KG system, reflecting pH-dependent differences in aggregation and particle growth. The PM group (native pH ≈ 6.0) showed a dominant sharp peak in the micron range (≈3–6 μm) and a secondary population around 50–100 μm. At pH 3.5, particles remained mainly in the few-micrometer range, but the distribution broadened toward larger sizes, suggesting rapid electrostatic complexation producing small primary aggregates along with some secondary association [9,26]. At pH 4.0–4.5, the distribution shifted toward larger particle sizes, generally tens to around one hundred micrometers, indicating more extensive growth and association of coacervate domains. Hydrogen bonding may also contribute to particle morphology and growth in this range [8]. At pH 5.0–5.5, near the gelatin isoelectric point (≈4.8–5.0), particles were dominated by hundreds to thousands of micrometers, reflecting extensive flocculation and formation of large, loosely packed aggregates. Near-neutral conditions (PM) favored smaller particles in the micron range due to weaker electrostatic interactions. Overall, these results show that pH strongly influenced aggregation. The intermediate pH range (4.0–4.5) produced moderately sized, relatively uniform particles, whereas higher-pH groups formed larger, loosely packed aggregates. Combined with SEM, rheology, extrusion, and Young’s modulus data, this suggests that pH regulation during KG preparation, especially at pH 4.0–4.5, produced a structural state that may be favorable for balancing flow during extrusion and post-deposition support after incorporation into the fruit matrix. However, particle-size data alone are not sufficient to establish a definitive structure–property relationship and should be regarded as a reasonable inference [24].

### 2.7. Possible Structural Interpretation of the pH-Dependent Printing Behavior

**Figure 9:**
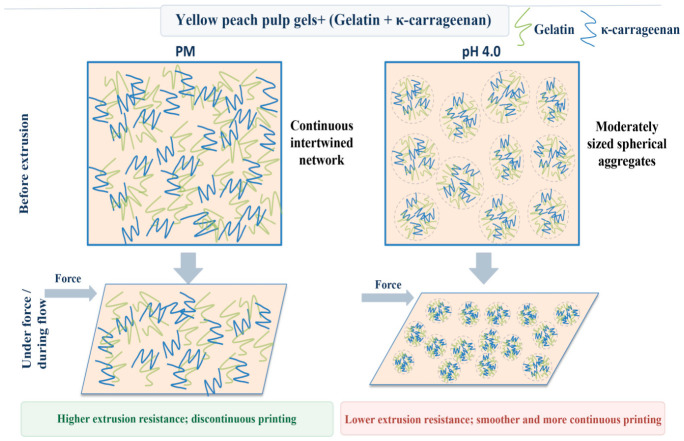
Schematic illustration of a possible structural interpretation for the different printing behaviors. The PM system is depicted as a relatively continuous intertwined structure, whereas the pH 4.0 system is illustrated as containing dispersed aggregates within the surrounding matrix. This schematic is intended to summarize a plausible interpretation of the observed trends rather than a definitive structural model.

A possible explanation for the different printing behaviors is that physical mixing and post-mixing pH regulation produced distinct structural states in the gelatin–κ-carrageenan system. Figure 9 provides a simplified schematic to summarize this interpretation, emphasizing that it represents a conceptual model rather than direct experimental evidence. In the physically mixed system, gelatin and κ-carrageenan may form a more continuous and intertwined network through direct blending, chain entanglement, and gelation. Such a structure can increase overall rigidity but may also elevate resistance to deformation and flow during extrusion.

By contrast, post-mixing pH regulation may promote electrostatic association between gelatin and κ-carrageenan, leading to the formation of composite aggregates dispersed within the surrounding matrix rather than a fully continuous polymer network. Under suitable pH conditions, this organization can facilitate rearrangement under applied stress while retaining mechanical support after deposition. In the present study, the improved balance between flowability and structural stability observed at pH 4.0 is consistent with the presence of moderately sized and relatively well-distributed aggregates, as illustrated schematically in Figure 9.

## 3. Conclusions

This study investigated the effect of post-mixing pH regulation of gelatin–κ-carrageenan on the structure and 3D printing performance of yellow peach pulp gels. The results showed that the structural state generated during KG preparation was closely related to extrusion behavior, mechanical support, and shape retention of the final PKGG inks. Among all formulations, the KG prepared at pH 4.0 showed the best overall printability. Although its pore-area fidelity was slightly lower than that of pH 3.5, it produced more stable multilayer cylinders and better-defined lattice structures, indicating a better balance between fine-path deposition and self-support. This sample also showed the lowest and most stable extrusion force and the highest Young’s modulus. SEM and particle-size results suggested that pH regulation changed the aggregation state and local morphology of the gelatin–κ-carrageenan system. The pH 4.0 treatment appeared to produce a more suitable structural state for continuous extrusion and post-deposition support, whereas higher-pH samples formed larger and less uniform aggregates, leading to poorer extrusion stability and shape retention. It should be noted that the mechanism proposed here is based mainly on indirect evidence. In addition, the PM control was maintained at its native pH, and the final pH values of the formulated PKGG inks were not directly measured. Therefore, the observed effects should be attributed to the combined influence of KG pre-regulation, structural rearrangement during preparation, and the acidic fruit-matrix environment. Future studies could include pH-adjusted PM controls, final ink pH measurements, and additional molecular-level characterization. Overall, post-mixing pH regulation of gelatin–κ-carrageenan may be a practical strategy for tuning fruit-based gel inks for 3D food printing.

## 4. Materials and Methods

### 4.1. Experimental Materials and Sample Preparation

κ-Carrageenan and gelatin (CAS: 9000-70-8) were purchased from Aladdin Biochem Technology Co., Ltd. (Shanghai, China). Glacial acetic acid (CAS: 64-19-7), diluted to a concentration of 1 mol/L, was sourced from Tianjin Damao Chemical Reagent Factory (Tianjin, China). Freeze-dried yellow flesh peach powder was obtained from Wanxing Biotechnology Co., Ltd. (Linyi, Shandong, China). All reagents were of analytical grade.

Gelatin–κ-carrageenan composite systems were prepared as follows. κ-Carrageenan (2%, *w*/*w*) and gelatin (2%, *w*/*w*) were first dissolved separately in deionized water at 90 °C and 50 °C, respectively, with continuous stirring until completely dissolved. The two solutions were then mixed at a 1:1 ratio and stirred in a constant-temperature magnetic stirrer (LICHEN, DF-101S, Shanghai, China) at 60 °C and 1200 rpm for 20 min. After mixing, the pH of the dispersion was measured using a pH meter (FiveEasy Plus, Mettler Toledo, Shanghai, China) and adjusted to 3.5, 4.0, 4.5, 5.0, or 5.5 with 1 mol/L acetic acid. After pH adjustment, the dispersions were stirred for an additional 2 min before storage. The pH-adjusted mixtures were regarded as the KG groups. In contrast, the physical mixture (PM) control was prepared by mixing gelatin and κ-carrageenan at the same concentrations without pH adjustment, and its native pH was approximately 6.0. It should be noted that using only the PM at its native pH does not allow a clear distinction between the effects of pH adjustment and complex coacervation; future studies could include pH-adjusted PM controls to separate these effects. All dispersions were stored at 4 °C and returned to room temperature before subsequent processing.

### 4.2. Spray-Drying Microencapsulation

The pH-adjusted gelatin–κ-carrageenan dispersions (approximately 500 g per batch) were spray-dried using a Mini Spray Dryer (BUCHI, B-290, Flaville, Switzerland) operated at 100% aspirator capacity. The drying process was conducted at an inlet temperature of 160 °C, an outlet temperature of 107 °C, and a pump setting of 10%, following the method outlined by [27] with slight modifications. The total spray-drying time for each batch was approximately 1.5 h. The final moisture content of the resulting KG powder was about 5%, as determined by a standard moisture analyzer. The powder was collected, sealed, and stored at room temperature (25 °C) for further use.

### 4.3. Sample Preparation for 3D Printing

Freeze-dried yellow-flesh peach powder, water, and gelatin–κ-carrageenan complex coacervates were blended at a weight ratio of 8:15:1 (*w*/*w*/*w*). The mixture was homogenized by thorough stirring at 1200 rpm to obtain a uniform dispersion, and then allowed to hydrate overnight at room temperature (25 °C). The pH adjustment was carried out during preparation of the gelatin–κ-carrageenan system before spray drying, rather than after incorporation into the yellow peach pulp matrix. The resulting dispersion, containing yellow peach powder, water, and either KG or PM, was referred to as PKGG and used for subsequent 3D printing experiments. In this study, the pH adjustment was applied to the KG system before incorporation into yellow peach pulp. Therefore, the pH labels of pH 3.5–5.5 refer to the pre-regulated pH of KG rather than the measured final pH values of the formulated PKGG inks. This design aimed to evaluate whether pH-regulated KG could function as an additive-like material to improve the printability of fruit-based inks after mixing with the pulp matrix.

### 4.4. 3D Printing Experiments

3D printing experiments were conducted using an extrusion-based 3D food printer (SHINNOVE-D1, Hangzhou Shiyin Technology, Hangzhou, China). All prints were performed at room temperature (25 °C) using a 0.8 mm nozzle, a layer height of 1.0 mm, an infill density of 95%, and a printing speed of 30 mm/s. The printer was operated using Simplify3D software (version 4.0.1). All reported printing results were obtained under this single set of conditions. Although the selected layer height (1.0 mm) slightly exceeds the nozzle diameter (0.8 mm), this choice was guided by the rheological behavior of the pH 4.0 ink and established extrusion printing principles. Extrusion outcomes in 3D food printing are fundamentally controlled by the balance between shear-induced flow within the nozzle and rapid structural recovery upon deposition. Materials exhibiting moderate viscoelasticity, finite yield stress, and shear-thinning behavior can undergo slight expansion (extrudate swell) upon exit from the nozzle, promoting interlayer contact and adhesion despite small geometric mismatches between nozzle diameter and layer height [2,28].

Print performance was evaluated by printing 20 mm × 20 mm × 4 mm squares with holes (6 mm × 6 mm × 4 mm) and hollow cylinders (φ20 mm × 70 mm). Pore areas of the printed samples were measured using ImageJ (version 1.52a). Print stability was assessed based on the height of the hollow cylinders and the Young’s modulus. Printing fidelity was calculated using the formula:

Printing fidelity = (1 − ∣Printed area − Designed area∣/Designed area) × 100%

### 4.5. Rheological Property Determination

The rheological characteristics of PKGG were determined using an Anton Paar Rheometer (Paar physica MCR 102, Anton Paar Company, Ostfildern, Germany) equipped with a parallel plate geometry (Ø25 mm, 1 mm gap). All the tests were conducted at 25 °C [19].

The viscosity sweep test was performed over a shear rate range of 0.1 to 1000 s^−1^. The measured viscosity values at different shear rates were subsequently fitted to the power-law model as follows:

η=Kγn

where *η* is the dynamic viscosity (Pa⋅s), K is the consistency index (Pa⋅s^n^), *γ* is the shear rate (s^−1^) and *n* is the flow behavior index [29].

The dynamic viscoelastic properties of every sample were assessed over an angular frequency range of 0.1–100 rad/s at 1% strain (fell within the linear viscoelastic region), according to the method described by [15]. The storage modulus (G′) and the loss modulus (G″) of the sample were both measured.

The minimum flow stress value of a sample was identified using an oscillation test, following the method reported by [30], at shear stresses ranging from 1 to 800 Pa with a fixed oscillation frequency of 1 Hz. The curves of G′ and loss modulus G″ under different shear stresses are recorded, and the intersection point is the minimum flow stress point.

### 4.6. Determining the Simulated Extrusion Capability of 3D Printing

The simulated piston-extrusion performance relevant to extrusion-based 3D food printing was evaluated using a texture analyzer (TA-XT Plus, Stable Micro Systems Ltd., Godalming, UK) equipped with a flat-ended probe (P/36R) and a custom-built extrusion simulation module. Each sample was transferred into a 60 mL syringe-type cartridge to emulate the printing barrel, and the cartridge was fixed in an external sleeve/holder to ensure coaxial alignment and minimize lateral movement during testing. In this configuration, the probe served as the piston rod, whereas the texture analyzer provided the driving motion analogous to the motor-controlled piston of a piston-based 3D food printer. The test sequence was performed under a compression–extrusion program with a pre-test speed of 1.0 mm/s, a test speed of 0.02 mm/s (selected to match the piston movement rate used during printing), and a post-test speed of 1.0 mm/s. The trigger force was set to 5.0 g, and the extrusion distance was fixed at 5 mm. Force data were acquired continuously throughout the run, generating a force–time profile that reflects the evolution of piston resistance during extrusion. For subsequent comparison among formulations, the steady-state extrusion force was extracted from the plateau region of the force–time curve, where the signal became relatively stable after the initial transient increase.

### 4.7. Young’s Modulus Determination

The Young’s modulus of PKGG was measured by a compression test with a texture analyzer (TA-XT. Plus, Stable Micro Systems Ltd., Godalming, UK) fitted with a flat probe (P/36R). Each sample (5 g) was placed in a beaker and subjected to compression testing. The test parameters were as follows: pretest speed = 2.0 mm/s; test speed = 2.0 mm/s; posttest speed = 2.0 mm/s; trigger type: button; compression distance = 3 mm. The stress–strain curve was recorded, and the slope of the 2–5% strain was taken as the Young’s modulus [31].

### 4.8. Particle Size Distribution of KG

The procedure was adapted from [32] with modifications; the particle size distribution of KG was measured by a laser diffraction particle size analyzer (Malvern Panaco, mastersizer 3000, Malvern, UK). The sample is first shaken completely and then added drop by drop to a mixing tank filled with deionized water until the obscuration reaches approximately 20%. Measurements were repeated 3 times for each sample.

### 4.9. SEM Morphology

The surface morphology of the powder was observed by scanning electron microscopy (SEM, E-1045, Hitachi Corporation, Tokyo, Japan) [33]. Prior to imaging, the samples were gold-coated using a sputter coater under the following conditions: accelerating voltage 15 kV, sputtering current 20 mA, sputtering time 30 s, working distance 10 mm, and sputtering chamber pressure 20 Pa. SEM images of KG were acquired at an accelerating voltage of 5 kV under low-vacuum conditions, with a working distance of 10 mm and a representative field of view at 2000× magnification.

Morphological microstructural observations of PKGG were conducted following the method described by [34] with minor modifications. Samples were frozen at −80 °C for 12 h, followed by freeze-drying using a laboratory-scale freeze dryer at a condenser temperature of −50 °C and a chamber pressure of 50 Pa. The freeze-dried samples were cut into small pieces measuring 5 mm × 5 mm × 2 mm and examined by scanning electron microscopy (Apreo TM 2 SEM, Thermo Fisher Scientific, Waltham, MA, USA) at an accelerating voltage of 5 kV, low-vacuum mode, a working distance of 10 mm, and a magnification of 1000×.

### 4.10. Statistical Analysis

The experimental data were analyzed using one-way analysis of variance (ANOVA), with results presented as means ± standard deviations. All experiments were conducted in triplicate. Duncan’s multiple range test was applied to determine significant differences among mean values at a 95% confidence level. Statistical analyses were performed using SPSS Statistics (version 19, SPSS, Chicago, IL, USA). Dynamic viscosity was fitted using the power-law model, with model fitting conducted in OriginPro 2021 (OriginLab, Northampton, MA, USA).

## Data Availability

The original contributions presented in this study are included in the article. Further inquiries can be directed to the corresponding author.
